# High-Resolution Vessel Wall Magnetic Resonance Imaging of Small Unruptured Intracranial Aneurysms

**DOI:** 10.3390/jcm10020225

**Published:** 2021-01-10

**Authors:** Łukasz Zwarzany, Ernest Tyburski, Wojciech Poncyljusz

**Affiliations:** 1 Department of Diagnostic Imaging and Interventional Radiology, Pomeranian Medical University, Unii Lubelskiej 1, 71-252 Szczecin, Poland; wponcyl@poczta.onet.pl; 2 Institute of Psychology, SWPS University of Social Sciences and Humanities, Kutrzeby 10, 61-719 Poznań, Poland; etyburski@swps.edu.pl

**Keywords:** unruptured intracranial aneurysm, aneurysm wall enhancement, vessel wall imaging, MRI

## Abstract

*Background*: We decided to investigate whether aneurysm wall enhancement (AWE) on high-resolution vessel wall magnetic resonance imaging (HR VW-MRI) coexists with the conventional risk factors for aneurysm rupture. *Methods*: We performed HR VW-MRI in 46 patients with 64 unruptured small intracranial aneurysms. Patient demographics and clinical characteristics were recorded. The PHASES score was calculated for each aneurysm. *Results*: Of the 64 aneurysms, 15 (23.4%) showed wall enhancement on post-contrast HR VW-MRI. Aneurysms with wall enhancement had significantly larger size (*p* = 0.001), higher dome-to-neck ratio (*p* = 0.024), and a more irregular shape (*p* = 0.003) than aneurysms without wall enhancement. The proportion of aneurysms with wall enhancement was significantly higher in older patients (*p* = 0.011), and those with a history of prior aneurysmal SAH. The mean PHASES score was significantly higher in aneurysms with wall enhancement (*p* < 0.000). The multivariate logistic regression analysis revealed that aneurysm irregularity and the PHASES score are independently associated with the presence of AWE. *Conclusions*: Aneurysm wall enhancement on HR VW-MRI coexists with the conventional risk factors for aneurysm rupture.

## 1. Introduction

Subarachnoid haemorrhage (SAH) from aneurysm rupture is a devastating condition with high mortality and morbidity rates [1,2]. To prevent this life-threatening event, unruptured intracranial aneurysms (UIA) are managed either surgically or endovascularly. Despite the technological advances in both methods of treatment, they pose a risk of serious complications [3]. Appropriate patient selection is fundamental to ensure that the benefits of aneurysm repair outweighs the risk of treatment.

In patients with UIA, therapeutic decisions are made based on the assessment of aneurysm rupture risk. Several attempts have been made to develop a method for estimating the risk of intracranial aneurysm rupture. The PHASES score was created to predict a patient’s 5-year absolute risk of aneurysm rupture on the basis of six patient and aneurysm characteristics (population, hypertension, age, size of aneurysm, earlier SAH from another aneurysm, site of aneurysm) [4]. However, a significant proportion of ruptured aneurysms would have a low risk of rupture according to the PHASES score when evaluated retrospectively [5]. This is especially true for small aneurysms, which account for an increasing majority of aneurysmal SAH [6]. Thus, new prognostic factors for aneurysm rupture are undoubtedly needed.

High-resolution vessel wall magnetic resonance imaging (HR VW-MRI) opens up new possibilities in the diagnostic work-up of cerebrovascular diseases [7]. Aneurysm wall enhancement (AWE) on HR VW-MRI is presumed to be an imaging marker of aneurysm instability, which may indicate lesions at higher risk of rupture [8,9,10]. However, there is insufficient evidence to support routine use of this new imaging tool in the decision-making for UIA treatment.

Due to the relatively low risk of rupture of small intracranial aneurysms, clinicians are often faced with the dilemma of whether to treat patients with such lesions or to manage them conservatively. Thus, better understanding of AWE on HR VW-MRI may provide useful insights in patient selection for aneurysm repair. In this study, we investigated the prevalence of AWE on HR VW-MRI in small UIA and whether it is associated with conventional risk factors for aneurysm rupture.

## 2. Materials and Methods

### 2.1. Patients

This study was approved by a local ethics committee (KB-0012/112/19). We prospectively collected adult patients with small UIA (defined as UIA with a maximum diameter ≤10 mm) who underwent HR VW-MRI at our institution (Department of Diagnostic Imaging and Interventional Radiology, Pomeranian Medical University, Szczecin, Poland) between June 2019 and September 2020. Written informed consent prior to MRI scanning was obtained from all patients. Patient demographics and clinical characteristics were recorded, including sex, age, family history of intracranial aneurysm, presence of multiple intracranial aneurysms, prior aneurysmal SAH, smoking status (current smoker, previous smoker, or never smoked), hypertension (defined as systolic blood pressure ≥140 mm Hg, diastolic blood pressure ≥90 mm Hg, or the use of antihypertensive medication), diabetes mellitus, and daily aspirin intake.

### 2.2. Imaging Protocol and Analysis

HR VW-MRI was performed on a 3.0T MRI system (Signa Pioneer; GE Healthcare, Milwaukee, WI, USA) using a 21-channel head/neck coil. The imaging protocol included three-dimensional (3D) time of flight MR angiography (TOF MRA), pre- and post-contrast 3D T1-weighted (T1W) fast spin-echo (FSE) sequence with variable refocusing flip angles (CUBE; GE Healthcare). The acquisition parameters of 3D T1W FSE sequence were as follows: repetition time (TR)/echo time (TE) = 604 ms/minimum; field of view (FOV) = 180 × 180 mm; matrix = 224 × 224; bandwidth = 50.0 kHz; echo train length (ETL) = 30; spatial resolution = 0.8 × 0.8 × 0.8 mm (interpolated to 0.8 × 0.8 × 0.4 mm); number of excitations (NEX) = 4. Gadoterate meglumine (Dotarem; Guerbet LLC, Villepinte, France) was administrated intravenously (0.1 mmol/kg), and 3D T1W FSE sequence was repeated with a 5-min delay after contrast agent injection.

The images were reviewed by two radiologists who reached decisions by consensus. The readers were free to change the window settings and the zoom level as necessary. They were allowed to use multiplanar reconstruction (MPR) as well as maximum intensity projection (MIP), minimum intensity projection (MinIP) and 3D reconstructions. For the TOF MRA images, the evaluation criteria were as follows: aneurysm location, aneurysm size (defined as the largest aneurysm diameter in any direction), aneurysm neck width, and aneurysm irregularity (defined as having lobulated contour or daughter sacs). The dome-to-neck ratio was calculated and used to identify wide-necked aneurysms (e.g., dome-to-neck ratio ≤ 2). Pre- and post-contrast 3D T1W FSE images were reviewed to determinate the presence or absence of AWE. We used a broad definition of AWE, including either thin or thick, partial or circumferential enhancement, as well as focal eccentric enhancement. The PHASES score was calculated for each aneurysm as described in the paper by Greving et al. [4]. In patients with multiple small UIA, each aneurysm was included and evaluated independently.

### 2.3. Statistical Analysis

Statistical analysis was done using the IBM SPSS 25 Statistical package (IBM Corp, Redmont, VA, USA). Continuous variables are presented as mean and standard deviation (*SD*), while nominal variables are presented as number and percent (%). The normality of the distribution of continuous variables was tested with the Shapiro-Wilk test. The differences in proportions between the two groups (UIA with and without wall enhancement) were analysed using the independent sample Student’s *t*-test (for continuous variables with normal distribution), the chi-square test for cross-tabulation (for two nominal variables) with Yates continuity correction for 2 × 2, and the chi-square test for cross-tabulation (for more than two nominal variables) with Bonferroni correction. Multivariate logistic regression (backward elimination, the Wald chi-square test) was further performed to identify independent risk factors of AWE (with the Hosmer-Lemeshow test for evaluating the goodness of fit of logistic regression models). The alpha criterion level was set at 0.05 in all statistical analyses.

## 3. Results

### 3.1. Patient and Aneurysm Characteristics

Patient and aneurysm characteristics are shown in Table 1. This study included 46 patients (39 females and 7 males) aged from 26 to 78 years, with a mean age of 58.6 and a standard deviation of 13.4 years. Seven patients (15.2%) had a family history of intracranial aneurysm, 5 (10.9%) had a prior SAH from another aneurysm, 23 (50.0%) had hypertension, 6 (13.0%) had diabetes mellitus, 12 (26.1%) took aspirin daily, 9 (19.6%) were current smokers, 17 (37.0%) were previous smokers, and 20 (43.5%) have never smoked. Multiple aneurysms were diagnosed in 19 (41.3%) patients and overall, 64 aneurysms were included for the analysis.

The aneurysm size ranged from 2.5 to 8.8 mm (mean 4.6 mm). Fourteen (21.9%) aneurysms presented with irregular shape. Fifty-four (84.4%) aneurysms were located in the anterior circulation, other 10 (15.6%) were in the posterior circulation.

### 3.2. Aneurysm Wall Enhancement and Relevant Factors

**Figure 1:**
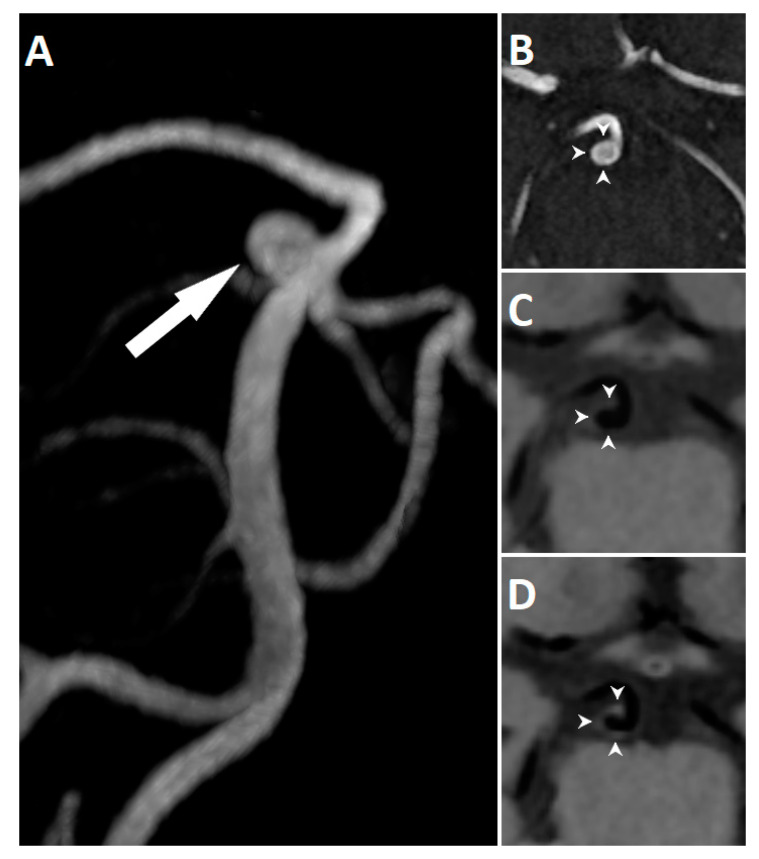
3D reconstruction of TOF MRA showing an unruptured basilar tip aneurysm (arrow) in a 60-year-old woman (**A**). TOF MRA (**B**) and corresponding high-resolution vessel wall sequence before (**C**) and after (**D**) contrast agent administration. The aneurysm demonstrates circumferential wall enhancement (arrowheads).

Of the 64 aneurysms, 15 (23.4%) showed wall enhancement on post-contrast HR VW-MRI. A representative case of AWE is presented in Figure 1.

**Figure 2:**
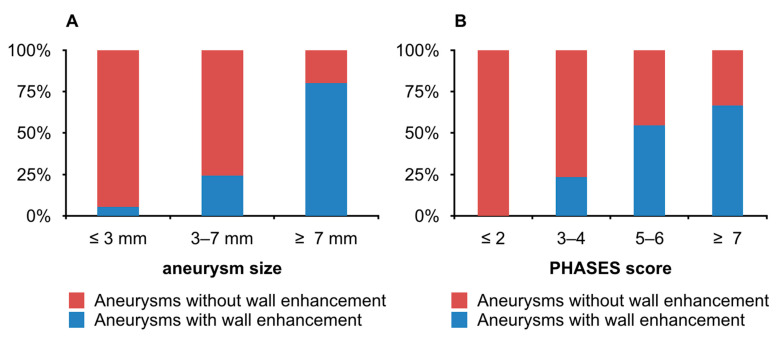
Differences in proportions of aneurysms with wall enhancement among different aneurysm size groups (**A**) and among different PHASES score groups (**B**).

The mean aneurysm size in the AWE group was significantly larger than in the non-AWE group (*p* = 0.001). The percentage of aneurysms with wall enhancement increased with the aneurysm size (Figure 2A). The differences in the frequency of AWE reached statistical significance in aneurysms ≤3 mm—5.6% (1/18; *p* < 0.05) and in aneurysms >7 mm—80.0% (4/5; *p* < 0.05). There were significantly more wide-necked aneurysms in the non-AWE group (79.6% vs. 40% in the AWE group; *p* = 0.009). The proportion of aneurysms with irregular shape in the AWE group was 57.1% (8/15), which was significantly higher than 42.9% (6/49) in the non-AWE group (*p* = 0.003). There were no significant differences between the two groups in the distribution of aneurysm location and the presence of multiple aneurysms.

The percentage of aneurysms with wall enhancement increased with the PHASES score (Figure 2B). The differences in the frequency of AWE reached statistical significance in aneurysms with the PHASES score ≤ 2—0.0% (0/20; *p* < 0.05) and in aneurysms with the PHASES score 5–6—54.6% (6/11; *p* < 0.05). There was a significant age difference between the two groups of patients (*p* = 0.011). Moreover, AWE was more frequently found in patients with prior SAH from another aneurysm (26.7% vs. 4.1%; *p* = 0.034). The distribution of sex, family history of intracranial aneurysm, smoking status, hypertension, diabetes mellitus, and daily aspirin intake showed no statistical differences.

Multivariate logistic regression was performed to identify the independent factors associated with AWE using a backward elimination process (Table 2). Finally, the PHASES score (*p* = 0.003) and aneurysm irregularity (*p* = 0.013) were significant predictors of AWE, explaining 50.0% of the variance (the model was well suited to the analysed data, *H* = 8.84; *p* = 0.356). Other independent variables (age, history of previous SAH from another aneurysm, aneurysm size, and dome-to-neck ratio) were excluded from the statistical model.

## 4. Discussion

According to the results of the International Study of Unruptured Intracranial Aneurysms (ISUIA), anterior circulation aneurysms smaller than 7 mm have a minimal risk of rupture [11]. However, in clinical practice, a significant proportion of patients presenting with aneurysmal SAH are diagnosed with small ruptured aneurysm [12]. This is due to the fact that small aneurysms are much more prevalent than large aneurysms. Thus, there is an undoubted need for new biomarker of aneurysm instability, which will be able to identify small aneurysms at higher risk of rupture.

Aneurysm wall enhancement on HR VW-MRI is suggested to represent inflammatory changes in the aneurysm wall. This hypothesis has been supported by a few studies with histopathological correlation [10,13,14,15,16]. Larsen et al. reported that AWE on HR VW-MRI is associated with inflammatory cell infiltration, neovascularisation, and the presence of vasa vasorum [14]. None of these histopathological findings were found in aneurysms without wall enhancement. In another study by Quan et al., specimens of aneurysms with wall enhancement showed high expression of inflammatory markers [16]. Although the exact mechanics are unknown, inflammation is believed to play an important role in the aneurysm formation, growth, and rupture [17]. Therefore, AWE on HR VR-MRI might be a potential radiological marker of aneurysm instability. In present study, we decided to investigate whether AWE on HR VW-MRI coexists with the conventional risk factors for aneurysm rupture.

Aneurysm size is a commonly accepted risk factor for aneurysm rupture [11,18]. In our study, the mean aneurysm size was 4.6 mm. We observed wall enhancement in 23.4% of the aneurysms, which is similar to other studies investigating small UIA [19,20,21]. In the study by Backes et al., which included predominantly small UIA, 29% of the aneurysms manifested AWE [19]. The authors stated that the aneurysm size is the strongest determinant of AWE. In general, data from the literature indicates that larger aneurysms are more likely to show wall enhancement [20,21,22]. Liu et al. found wall enhancement in all of the aneurysms larger than 13 mm, but in only 12% of the aneurysms smaller than 7 mm [20]. In our study, there was a statistically significant difference in the mean aneurysm size between aneurysms with and without wall enhancement. With increasing aneurysm size, the proportion of aneurysms with wall enhancement increased progressively.

Dome-to-neck ratio is also an important morphological feature of aneurysm to evaluate. Higher dome-to-neck ratios are observed in ruptured compared to unruptured aneurysms [23]. Interestingly, we observed that aneurysms with wall enhancement had a significantly higher mean dome-to-neck ratio.

Previous studies reported different findings regarding the association between AWE and aneurysm irregularity [20,21], which is another recognizable risk factor for aneurysm rupture [24,25]. Significantly more aneurysms with wall enhancement had an irregular shape than aneurysms without wall enhancement. Furthermore, the multivariate logistic regression analysis revealed that aneurysm irregularity is independently associated with the presence of AWE.

**Figure 3:**
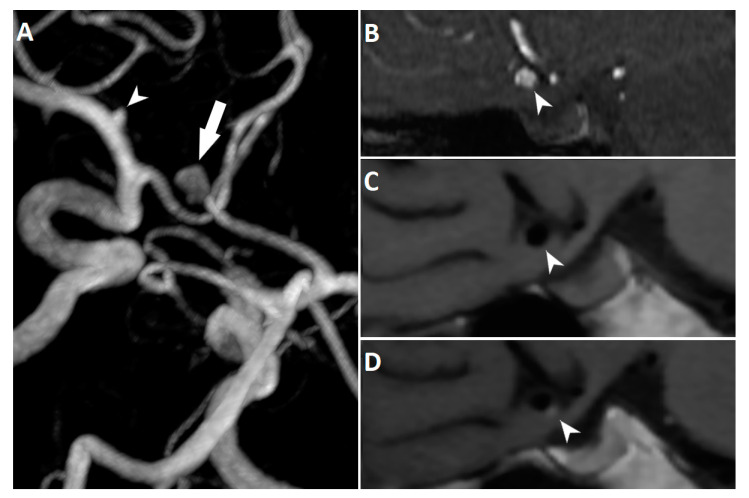
3D reconstruction of TOF MRA showing an unruptured anterior communicating artery aneurysm (arrow) in a 65-year-old woman. The aneurysm has an irregular shape with a bleb at the inferior border. Small left middle cerebral artery aneurysm (arrowhead) is also noted (**A**). TOF MRA (**B**) and corresponding high-resolution vessel wall sequence before (**C**) and after (**D**) contrast agent administration. The aneurysm demonstrates focal wall enhancement at the bleb (arrowheads).

It has been shown previously that AWE might have focal or circumferential pattern. According to Edjlali et al., a thick circumferential pattern of AWE has the highest specificity for differentiating between stable and unstable aneurysms [9]. Due to the relatively small number of patients included in our study, we decided not to discriminate between focal or circumferential pattern of AWE in the statistical analysis. However, we encountered both types of AWE when evaluating the images (Figure 3). Several studies have investigated the relationship between focal AWE and specific hemodynamic factors [26,27,28]. The results of these studies revealed that focal AWE occurs at the location of lower wall shear stress, a hemodynamic condition that may be related to a higher risk of aneurysm rupture. In their recent paper, Cebral et al. reported that low wall shear stress is associated with atherosclerotic and hyperplastic changes in the aneurysmal wall [29]. In one of the abovementioned studies, Larsen et al. provided the results of the histopathological analysis from a subset of patients who underwent microsurgical aneurysm clipping [26]. The presence of histological inflammatory markers was significantly associated with a larger extent of AWE. Interestingly, aneurysms with a larger enhancement area had significantly higher PHASES scores.

Data from the literature shows that small aneurysms are at higher risk of rupture if the patient had a prior SAH from another aneurysm [12]. In our study, aneurysms of patients with a prior aneurysmal SAH presented wall enhancement more frequently. Moreover, we also found that patients harbouring aneurysms with wall enhancement were significantly older. This is on the contrary to the findings of Liu et al., who reported that age is inversely associated with AWE [20]. In the present study, all of the other patient characteristics did not show significant differences between the two groups. However, a recent study has found that the use of aspirin daily for ≥6 months significantly decreases AWE [22]. This might be explained by its anti-inflammatory properties. It is worth emphasizing that all patients included in our study were asymptomatic. An interesting study was carried out by Fu et al., who found that AWE is more frequently observed in symptomatic than in asymptomatic patients with UIA [30]. In addition, the authors reported that AWE was the only independent factor associated with symptoms in the multivariable logistic regression analysis. From 46 patients in our study, 39 (85%) were female and therefore dominate the database. This high percentage of female patients does not truly represent the disease’s nature and should be considered a study limitation.

Finally, we calculated the PHASES score for each aneurysm and found that the mean PHASES score was significantly higher for aneurysms with wall enhancement than aneurysms without wall enhancement. With the increasing PHASES score, the proportion of aneurysms with wall enhancement increased progressively. Furthermore, we performed the multivariate logistic regression analysis, which revealed that the PHASES score is independently associated with the presence of AWE. Although the difference in the mean PHASES score between two groups (AWE and non-AWE) was almost twofold, 5-year absolute risk of aneurysm rupture was similar (1.3% and 0.7%, respectively). Study population of patients with small UIA might have contributed to this, as aneurysm size is the main factor affecting the PHASES score. Other studies also confirmed that wall enhancement is detected more frequently in aneurysms at higher risk of rupture by the PHASES score [21,31].

The major limitation of our study is its cross-sectional design. It is clear that only longitudinal studies can directly answer the question whether AWE is an imaging marker of aneurysm instability, identifying lesions at higher risk of growth or rupture. However, such studies can be difficult to perform as a high level of anxiety seen in patients diagnosed with intracranial aneurysm prompts the decision to treat them. Moreover, the annual rupture risk of small UIA is very low, which means that such studies require a large number of patients followed up for many years. Since the publication of the first paper describing AWE by Matouk et al. in 2013 [32], only three additional studies comparing the natural history of intracranial aneurysms with and without wall enhancement have been carried out [9,33,34]. A recent meta-analysis of these studies showed a high negative predictive value of AWE and aneurysm instability, indicating that aneurysms without wall enhancement are unlikely to rupture [35]. On the other hand, a low positive predictive value was found, meaning that a significant number of aneurysms demonstrating wall enhancement may actually be stable. Due to the differences in studies designs, caution is warranted when interpreting the findings of this meta-analysis. Firstly, Vergouwen et al. defined aneurysm instability as aneurysm growth or rupture [33]. In another study, unruptured symptomatic aneurysms were also classified as unstable [9]. Furthermore, there was discrepancy between the length of imaging follow-up. In the study by Edjlali et al., 276 stable aneurysms, which constituted about 75% of the total stable aneurysms included in the meta-analysis, were followed up for 6 months only [9]. It is a relatively short period in comparison with a median imaging follow-up of 27 months in the study by Vergouwen et al. [33]. According to the results of the meta-analysis, only two out of 349 patients experienced aneurysmal SAH during follow-up [35]. In one of these patients, an aneurysm rupture occurred 31 months after the baseline HR VW-MRI. Thus, larger samples and longer follow-up are warranted in future studies. In addition, there is also a noticeable discrepancy between the reported frequency of AWE. Tian et el. observed wall enhancement in 71% of the investigated aneurysms, all of which proved to be stable [34]. On the contrary, only 38.4% of the stable aneurysms in the study by Edjlali et al. showed wall enhancement [9]. Apart from the differences in patient and aneurysm characteristics, this might have been due to the lack of standardization of imaging protocols across studies. There is an ongoing multicentre clinical trial (called the UCAN Project) to investigate the predictive value of AWE for small UIA growth [36]. Thanks to the strictly defined inclusion/exclusion criteria and standardized imaging protocol, this study may provide the evidence we need to establish the role of AWE in the management of patients with small UIA.

In present study, we acquired HR VW-MRI scans with a spatial resolution of 0.8 × 0.8 × 0.8 mm (interpolated to 0.8 × 0.8 × 0.4 mm), which is comparable to other series [9,20,27]. It is also coherent with the minimal HR VW-MRI sequence parameters set by the UCAN Project Investigators [36]. More than a quarter of the aneurysms included in our study were ≤3 mm and the minimum aneurysm size was 2.5 mm. In our view, the quality of the acquired images was suitable enough to reliably evaluate all of these very small aneurysms. There was no case of severe motion artifacts degrading interpretation and limiting diagnosis of AWE. However, we cannot exclude with certainty that the lower incidence of wall enhancement in the aneurysms ≤3 mm was at least partially due to their reduced conspicuity when compared to larger aneurysms. With the increasing availability of 7T MRI scanners, which are capable of acquiring images with even higher spatial resolution and better tissue contrast, future HR VW-MRI studies may provide more reliable data on the wall enhancement of small UIA.

In our study, HR VW-MRI images were reviewed simultaneously by two radiologists and the decision regarding the presence or absence of AWE was made by consensus. Thus, we did not calculate inter-rater agreement, which is a study limitation. However, it was reported to be good or excellent in other studies [8,21,30]. We decided to perform a visual assessment of AWE, comparing pre- and postcontrast images. This might be a subjective procedure, but on the other hand it seems to be the most feasible method to implement in daily clinical practice. Other investigators developed more objective methods for the assessment of AWE on HR VW-MRI [37,38]. Roa et al. showed that aneurysm-to-pituitary stalk contrast ratio is the most reliable objective method to quantify AWE [37]. With this method, the researchers achieved similar results using scanners from different manufacturers as well as scanners of different magnetic field strengths.

The low positive predictive value of AWE and aneurysm instability may be partially explained by the insufficient slow-flow suppression by the currently available HR VW-MRI sequences. A phantom-based study by Cornelissen et al. showed that slow flow along the aneurysm wall mimics AWE [39]. Thus, caution must be taken when interpreting HR VW-MRI studies. This is especially true in the cases of large and irregular aneurysms, as these lesions often have complex flow. The authors also demonstrated a significant improvement in the performance of the slow-flow suppression when preparation pulses, such as motion-sensitized driven equilibrium (MSDE) and delay alternating with nutation for tailored excitation (DANTE), were used. We did not implement preparation pulses in our study. However, insufficient slow-flow suppression seems to be a minor issue in the cases of small aneurysms. Nevertheless, further development of these techniques is crucial for optimization of HR VW-MRI.

Although new data on AWE is constantly emerging, it still remains a controversial topic [40,41]. To better understand this imaging finding, corelation with histological analysis and hemodynamic conditions is warranted in future studies. This, unfortunately, may be difficult to achieve due to the increasing number of intracranial aneurysms treated by the endovascular approach.

## 5. Conclusions

Aneurysm wall enhancement is significantly more prevalent in aneurysms presenting morphological characteristics known to be associated with increased risk of aneurysm rupture. It is also more frequently observed in older patients, and those with a history of prior aneurysm SAH. Higher PHASES score and irregular shape of the aneurysm are independent factors associated with the presence of AWE. Our findings support the hypothesis that AWE on HR VW-MRI might be a radiological marker of aneurysm instability. However, more and larger longitudinal studies are needed to definitely elucidate this issue.

## Figures and Tables

**Table 1:** Characteristics of unruptured intracranial aneurysms with and without wall enhancement.

Variable	AWE (*n* = 15)	Non-AWE (*n* = 49)	*t*/χ^2^	*p* Value
Age: *M* (*SD*)	64.80 (6.03)	58.22 (13.73)	−2.63 ^a^	0.011
Sex: f/m	15/0	40/9	1.87 ^b^	0.172
Family history of IA: yes/no	2/13	11/38	0.16 ^b^	0.688
Prior SAH from another aneurysm: yes/no	4/11	2/47	4.49 ^b^	0.034
Smoking status:				
Current smoker: yes/no	6/9	10/39	1.42 ^b^	0.233
Previous smoker: yes/no	7/8	17/32	0.28 ^b^	0.594
Never smoked: yes/no	2/13	22/27	3.63 ^b^	0.057
Hypertension: yes/no	10/5	24/25	0.82 ^b^	0.365
Diabetes mellitus: yes/no	1/14	8/41	0.27 ^b^	0.605
Daily aspirin intake: yes/no	2/13	12/37	0.31 ^b^	0.577
PHASES: *M* (*SD*)	5.13 (1.73)	2.61 (1.88)	−4.63 ^a^	0.000
PHASES: ≤ 2/3–4/5–6/≥ 7	0 */7/6 */2	20 */23/5 */1	15.18 ^c^	0.002
Size: *M* (*SD*)	5.79 (1.83)	4.24 (1.36)	−3.55 ^a^	0.001
Size: ≤ 3 mm/3–7 mm/≥ 7 mm	1 */10/4 *	17 */31/1 *	12.14 ^c^	0.002
Dome-to-neck ratio: *M* (*SD*)	2.25 (0.83)	1.72 (0.76)	−2.31 ^a^	0.024
Dome-to-neck ratio: ≤ 2/> 2	6/9	39/10	6.83 ^b^	0.009
Irregularity: yes/no	8/7	6/43	9.07 ^b^	0.003
Location: anterior/posterior circulation	11/4	43/6	0.88 ^b^	0.347
Multiple aneurysms: yes/no	11/4	24/25	1.85 ^b^	0.173

^a^ Student’s *t* test. ^b^ chi squared test with Yates continuity correction. ^c^ chi squared test without Yates continuity correction * Differences in proportion after Bonferroni correction. IA = intracranial aneurysm; SAH = subarachnoid haemorrhage; AWE = aneurysm wall enhancement.

**Table 2:** Multivariate analysis of variables independently associated with aneurysm wall enhancement.

Variable	Odds Ratio	95% Confidence Interval	*p* Value
PHASES	2.34	1.33–4.16	0.003
Irregularity	7.95	1.54–41.01	0.013

## Data Availability

The data presented in this study are available on request from the corresponding author.
